# Subsidize consumers or service firm? The design of a government subsidy policy for an older adult care service supply chain

**DOI:** 10.3389/fpubh.2026.1890740

**Published:** 2026-07-22

**Authors:** Lei Liu, Chen Zhou

**Affiliations:** 1 School of Humanities, Southeast University, Nanjing, China; 2 School of Foreign Languages, Southeast University, Nanjing, China

**Keywords:** consumer subsidy policy, older adult care service, game theory, service subsidy policy, social welfare

## Abstract

**Background:**

To promote the development of older adult care services, a government can offer two types of subsidy programs: a consumer subsidy policy and a service subsidy policy. Choosing an appropriate subsidy policy to maximize social welfare is a critical decision for the government.

**Methods:**

We consider an older adult care service supply chain consisting of the government, a service firm, and heterogeneous older adult consumers, who are classified into economy-conscious and quality-conscious segments, and develop a game-theoretic model to analyze the optimal subsidy program.

**Results:**

The structure of the optimal subsidy policy depends on the price sensitivity coefficient of economy conscious consumers, the service cost, and the market share of such consumers. As the price sensitivity coefficient increases, the consumer subsidy policy exhibits an increasingly pronounced advantage over the service subsidy policy in enhancing consumer surplus, firm profit, and social welfare. In particular, when the price sensitivity coefficient is high, the consumer subsidy policy always achieves such a tripartite outcome.

**Conclusions:**

Consumer subsidy policy lowers the financial threshold for accessing older adult care services, thereby promoting health equity among the older population, and this improved affordability does not necessarily come at the expense of service quality. Therefore, governments should adopt the price-sensitivity coefficient of older adults as a core criterion in subsidy design, since it simultaneously guides the economically optimal policy and the instrument most congruent with public health goals.

## 1 Introduction

According to data from the United Nations Population Fund (UNFPA), the global proportion of the population aged 65 years or older reached 10% in 2025, far exceeding the 7% threshold that defines an “aging society” by UN standards, indicating that population aging has become a global trend. Against this backdrop, building an efficient and inclusive older adult care system has become an urgent task for social development worldwide. In response, many countries have introduced policies and regulations to underscore the leading role of governments in the development of older adult care services. For example, China issued the Opinions on Deepening the Reform and Development of Older Adult Care Services; the United Kingdom enacted The Care and Support Regulations 2025; and Japan implemented the Long-Term Care Insurance Act. All these measures emphasize the importance of government leadership and institutional guarantees.

The price and quality of older adult care services are critical factors influencing the development of the “silver economy.” However, in practice, many older adult care facilities struggle to achieve sustainable operations due to high operating costs and relatively low service quality. Meanwhile, although there is strong potential demand for home-based older adult care services among the older population, a significant gap exists between effective

demand and actual consumption behavior, constrained by factors such as insufficient ability to pay and uneven service quality. Government subsidies, as an important intervention tool, are expected to play a dual role in improving service quality and alleviating price burdens.

Currently, two common forms of government subsidies exist. The first is the consumer subsidy policy, which directly allocates subsidies to economically disadvantaged or care-dependent older adults to enhance their ability to pay. At the national level, the Ministry of Civil Affairs of China launched a nationwide older adult care service consumption voucher program on January 1, 2026, providing direct purchasing subsidies to disabled and semi-disabled seniors; by March 2026, the program had benefited over 1.05 million seniors and stimulated an estimated 11.5 billion yuan in older adult care consumption.^1^ At the local level, for instance, in Beijing, low-income seniors receiving subsistence allowances are granted RMB 300 per month, those from low-income families receive RMB 200, and seniors from families with special birth planning policies receive RMB 100. Such subsidies directly enhance the payment capacity and service accessibility of older adults, incentivizing demand to compel older adult care facilities to improve service quality, thereby increasing consumer surplus, particularly benefiting low-income seniors. However, subsidies may push up market prices, and part of the subsidy may be captured by facilities through price increases, reducing subsidy efficiency and weakening policy effectiveness.

The second is the service subsidy policy, which directly supports older adult care institutions to enhance service supply capacity and quality. Guided by the State Council's Opinions on Promoting the Construction of a Basic Eldercare Service System (2023) and the 2024 Guidelines to Boost the Silver Economy and Improve Welfare of the Older Adult,^2^ local governments have established bed-construction grants and operational subsidies tied to service quality and capacity for private older adult care institutions. For example, in Yiliang County, Yunnan Province, rated older adult care facilities receive operating subsidies–RMB 100,000 for three-star, RMB 200,000 for four-star, and RMB 300,000 for five-star facilities. Such subsidies directly lower operating costs, facilitate facility upgrades and service quality improvements, enhance the attractiveness of facilities, and expand service supply. In theory, they can increase consumer surplus and total social welfare. Nevertheless, service subsidies operate through an indirect welfare transmission mechanism: government funds are channeled to consumers via facilities. This entails the risk of subsidy leakage–facilities may simultaneously raise prices while improving quality, so that part of the subsidy is absorbed by facilities, and the actual benefits to consumers are diminished.

Intuitively, service subsidies and consumer subsidies act on the supply side and demand side of the older adult care market, respectively. The former helps improve service quality and long-term supply capacity but may weaken the benefit to consumers due to incomplete subsidy transmission. The latter directly increases seniors' payment capacity and consumption levels but may induce price increases and reduce subsidy efficiency. Hence, there is an inherent trade-off between the two types of subsidies in terms of service quality, price levels, and social welfare. How to select the optimal subsidy approach has thus become a key issue in the design of older adult care policies.

Most existing studies focused on the coordination and pricing of the older adult care service supply chain (1–4). For example, Zhao (1) studied channel coordination for uncertain demand in two-echelon older adult healthcare service supply chain, adopting a loss-sharing contract embedded with options to derive optimal service capacity and sales effort, ensuring supply stability. Zhao (2) extended the research to dual-channel older adult healthcare service supply chain to analyze the optimal decision-making for older adult service providers and service integrators. By comparing centralized, Bertrand and Stackelberg game scenarios, the optimal coordination structure has been identified. Zhao and Hou (5) explored supplier effort coordination in dual-channel smart older adult care service supply chains. They found benefit-sharing contracts maximize supply chain profits, and demand segmentation facilitates dual-channel differentiated services and win–win outcomes. Zhao and Hou (6) employed Hotelling and Stackelberg game models to describe the optimal decision-making of the dual-channel smart older adult service care supply chain, and used simulation to quantify key parameter impacts.

Government subsidy policies have been widely adopted across numerous industries—including green manufacturing (7–9), agriculture (10–12), and the fashion industry (13, 14)—and results consistently demonstrate their effectiveness in fostering industrial development and enhancing social welfare. Nowadays, a growing body of literature has begun to examine the role of subsidy policies in the older adult care service industry, investigating the design and effectiveness of alternative subsidy schemes with the aim of providing theoretical foundations and practical guidance for more effective policy formulation. Taking China as the background, Song et al. (15) confirmed that subsidies effectively motivate private older adult care provision, and argued that regional supply-demand mismatch should be the basis for differentiated subsidy design in aging areas. Dong et al. (16) analyzed supply-side (service companies) and demand-side (older adult customers) subsidies via a Nash game model to present the effects of government subsidies on the price, quality, and quantity of demand for older adult services under different subsidy policies. Zhao and Hou (6) constructed a dual-channel older adult care service supply chain coordination model with differentiated government subsidies. The results reveal that cooperative modes achieve higher optimal pricing and service levels. Meanwhile, government subsidies generate stronger incentive effects on offline service channels than online ones, which helps better satisfy the emotional needs of the older adult. Considering the government subsidy customer, He et al. (17) established a three-stage older adult care service supply chain model to explore the optimal decisions and market performance of the government, ESP and ESI under different channel power structures and government intervention to guide practice.

In summary, existing research on older adult care service supply chains has predominantly focused on coordination mechanisms and pricing strategies. While several studies have examined the role of government interventions, the comparative welfare implications of subsidizing service providers vs. consumers remain underexplored. More critically, prior literature typically treats government subsidies as exogenous parameters within a single subsidy regime, lacking systematic analysis and comparison of optimal subsidy policy design. This study addresses these gaps by examining the government's optimal subsidy policy design in older adult care service supply chains from a social welfare maximization perspective.

Beyond their economic implications, subsidy policies for older adult care services carry profound public health consequences. Access to affordable, high-quality care is increasingly recognized as a determinant of healthy aging, yet the older adult population is far from homogeneous in its ability to pay. Price-sensitive, lower-income older adults—modeled here as economy-conscious consumers—are disproportionately likely to forgo or delay necessary care when prices rise, thereby worsening health outcomes and widening care-access disparities. The design of subsidy policies is therefore not merely an economic problem, but a public health imperative: which scheme more effectively reaches the most vulnerable older adult while sustaining the long-term viability of care providers is directly relevant to national aging strategies and health system sustainability. To this end, we investigate the following research questions: (1) given consumer heterogeneity, how should a firm determine its service pricing and quality decisions under alternative subsidy policy, and how should the government optimally design its subsidy policy? (2) Which subsidy policy enables the government to achieve the socially optimal outcome? (3) How do alternative subsidy policies differentially affect firm profitability, consumer surplus, and social welfare?

To address these questions, we model an older adult care service supply chain comprising three key actors: the government, an older adult care service provider, and heterogeneous older adult consumers. The government chooses between a demand-side subsidy (targeting consumers) and a supply-side subsidy (targeting the service provider) to maximize social welfare. Consumers are segmented into two types based on preference heterogeneity: price-sensitive consumers, who prioritize affordability, and quality-sensitive consumers, who prioritize service quality. We employ a game-theoretic framework to characterize the strategic interactions among the government, the firm, and consumers. We derive the equilibrium subsidy levels, pricing, and quality decisions under each subsidy policy and conduct comparative analyses to assess their respective impacts on firm profit, consumer surplus, and social welfare.

## 2 Model setup

### 2.1 Problem description and assumptions

In this study, we examine the older adult care service supply chain consisting of the government, older adult care service firm (hereinafter referred to as the firm), and older adult consumers (hereinafter referred to as consumers). As the service provider, the firm improves its service quality by taking measures such as upgrading service equipment and enhancing caregiver skills. Let s denote the level service quality improvement. Referring to He et al. (17) and Zhao and Hou (6), the cost of service improvement is quadratically related to service quality, that is, the cost of service improvement is $\frac{ms^{s}}{2}$, where *m* represents the cost coefficient of quality improvement. A smaller *m* indicates a stronger capability of the firm to improve service quality, implying a lower cost to achieve a given level of quality enhancement. Let *p* denote the service price, and we assume the marginal cost of service is zero.

Consumers decide whether to purchase older adult care services based on the service quality and price. According to their economic conditions and preferences, we classify consumers into two types: economy-conscious consumers and quality-conscious consumers. This two-type segmentation is a deliberate modeling simplification. Although older adult consumers differ in income, age, health status, dependency level, residence, and family support, our study focuses on the two behavioral dimensions most directly related to subsidy design: price sensitivity and quality sensitivity. Economy-conscious consumers represent older adult individuals mainly constrained by affordability, whereas quality-conscious consumers represent those who place greater weight on service quality. This parsimonious classification keeps the model tractable while capturing the key demand-side mechanism through which alternative subsidy policies affect market outcomes. Furthermore, we assume that the two consumer groups share the same sensitivity to service quality, which is normalized to one. This assumption allows us to focus on heterogeneity in price sensitivity, the consumer characteristic most directly relevant to the subsidy-design problem. Although this specification abstracts from potential interactions between price and quality preferences, it provides a parsimonious benchmark that is consistent with the existing literature. However, economy-conscious consumers exhibit a price sensitivity θ_1_ greater than their quality sensitivity, whereas quality-conscious consumers exhibit a price sensitivity θ_2_ smaller than their quality sensitivity, i.e., θ_1_>1>θ_2_>0. Without loss of generality, we assume the total market size of consumers is M, with proportions α and 1−α for economy-conscious and quality-conscious consumers, respectively.

Following related older adult care service supply chain studies (2, 16, 17), we adopt linear demand functions to capture consumers' responses to service price and quality. This specification is analytically tractable and allows the marginal effects of price and quality to be clearly identified. The additive form reflects the assumption that price and quality affect demand independently, which helps isolate the mechanisms through which consumer subsidies and service subsidies influence market outcomes. Hence, the demand of the two consumer types as linear functions of price and quality: *D*_1_ = *Mα* − θ_1_*p* + *s*, and *D*_2_ = *M*(1 − α) − θ_2_*p*+*s*. The total demand is *D* = *M*−*p*(θ_1_+θ_2_)+2*s*. Correspondingly, following Arya et al. (18), the consumer surplus (CS) can be expressed as: $CS=\int_{0}^{D^{*}}\frac{D^{*}-D}{\theta_{1}+\theta_{2}}dD=\frac{{\left(D^{*}\right)}^{2}}{2\left(\theta_{1}+\theta_{2}\right)}$.

To foster the development of the older adult care industry, the government employs subsidy policies as a regulatory instrument. This paper considers two subsidy schemes grounded in real-world policy practice. The first is a consumer subsidy policy (Model C), under which the government provides a per-unit subsidy *t* exclusively to economy-conscious consumers. The second is a service subsidy policy (Model S), under which the government offers a quality-contingent subsidy *rs* to the older adult care service provider, where *r* represents the subsidy rate per unit of quality improvement.

In the older adult care context, government subsidy policies typically pursue two objectives: improving consumer surplus and stimulating industry growth by enhancing firm profitability. Accordingly, the government selects a subsidy scheme to maximize social welfare, anticipating the subsequent decisions of the firm and consumers. Social welfare is defined as the sum of consumer surplus and firm profit minus government expenditure.

The game unfolds in three stages. In the first stage, the government selects a subsidy policy and determines the optimal subsidy level: the per-unit consumer subsidy *t* under Model C, or the quality-based subsidy rate *r* under Model S. In the second stage, upon observing the government's decision, the firm sequentially determines its service quality level *s* and service price *p*. In the third stage, consumers make their purchasing decisions given the prevailing price, quality, and subsidy conditions.

### 2.2 Consumer subsidy policy (Model C)

Under the consumer subsidy policy, economy-conscious consumers receive a government subsidy *t* per unit of older adult care service purchased, while quality-conscious consumers do not receive any government subsidy. In this case, the demand functions of the two types of consumers are given, respectively, by $D_{1}^{C}=M\alpha -\theta_{1}\left(p-t\right)+s$ and $D_{2}^{C}=M\left(1-\alpha \right)-\theta_{2}p+s$. Based on these demands, the firm's profit can be expressed as $\pi^{C}=pD_{1}^{C}+pD_{2}^{C}-\frac{1}{2}ms^{2}$.

** Lemma 1**. *Given the consumer subsidy t, the optimal service price and service quality are* $p=\frac{m\left(M+t\theta_{1}\right)}{2m\left(\theta_{1}+\theta_{2}\right)-4}$ *and* $s=\frac{M+t\theta_{1}}{m\left(\theta_{1}+\theta_{2}\right)-2}$.

By anticipating the firm's responses as in Lemma 1, the government sets the optimal consumer subsidy *t* to maximize the social welfare $SW^{C}=\pi^{C}+CS^{C}-tD_{1}^{C}$, where $tD_{1}^{C}$ denotes the government expenditure under the consumer subsidy policy. To ensure that the firm's profit functions are concave and the cost of quality improvement is not so low, we require that $m>m_{1}=8/\left(3\theta_{1}+6\theta_{2}-\sqrt{5\theta_{1}^{2}+4\theta_{2}^{2}}\right)$.

**Proposition 1**. *Under the consumer subsidy policy, the optimal consumer subsidy, service price and service quality are* $t^{C}=\frac{M\left(8+5m^{2}\theta_{1}\left(\theta_{1}+\theta_{2}\right)-4m\left(3\theta_{1}+\theta_{2}\right)-4\alpha {\left(-2+m\left(\theta_{1}+\theta_{2}\right)\right)}^{2}\right)}{\theta_{1}\left(16-12m\left(\theta_{1}+2\theta_{2}\right)+m^{2}\left(\theta_{1}+\theta_{2}\right)\left(\theta_{1}+8\theta_{2}\right)\right)}$, $p^{C}=\frac{mM\left(-6+4\alpha +3m\theta_{1}+4m\theta_{2}-2m\alpha \left(\theta_{1}+\theta_{2}\right)\right)}{16-12m\left(\theta_{1}+2\theta_{2}\right)+m^{2}\left(\theta_{1}+\theta_{2}\right)\left(\theta_{1}+8\theta_{2}\right)}$, *and* $s^{C}=\frac{-2M\left(2\alpha -3\right)\left(m\theta_{1}-2\right)-4mM\left(\alpha -2\right)\theta_{2}}{16-12m\left(\theta_{1}+2\theta_{2}\right)+m^{2}\left(\theta_{1}+\theta_{2}\right)\left(\theta_{1}+8\theta_{2}\right)}$.

### 2.3 Service subsidy policy (Model S)

Under the service subsidy policy, the firm receives a government subsidy r for each unit of service quality improvement. In this case, the demand functions are $D_{1}^{S}=M\alpha -\theta_{1}p+s$ and $D_{2}^{S}=M\left(1-\alpha \right)-\theta_{2}p+s$. Based on these demands, the firm's profit is $\pi^{S}=pD_{1}^{S}+pD_{2}^{S}+rs-\frac{1}{2}ms^{2}$.

**Lemma 2**. *Given the service subsidy r, the optimal service price and service quality are* $p=\frac{mM+2r}{2m\left(\theta_{1}+\theta_{2}\right)-4}$ *and* $s=\frac{M+r\left(\theta_{1}+\theta_{2}\right)}{m\left(\theta_{1}+\theta_{2}\right)-2}$.

By anticipating the firm's responses as in Lemma 2, the government sets the optimal consumer subsidy *t* to maximize the social welfare *SW*^*S*^ = π^*S*^ + *CS*^*S*^ − *rs*, where *rs* denotes the government expenditure under the service subsidy policy. Similarly, we require that *m* > *m*_2_ = 3/(θ_1_ + θ_2_).

**Proposition 2**. *Under the service subsidy policy, the optimal service subsidy, service price and service quality are* $t^{S}=\frac{mM}{2m\left(\theta_{1}+\theta_{2}\right)-6}$, $p^{S}=\frac{mM}{2m\left(\theta_{1}+\theta_{2}\right)-6}$, *and* $s^{S}=\frac{3M}{2m\left(\theta_{1}+\theta_{2}\right)-6}$.

## 3 Model analysis

### 3.1 Comparison of service prices, quality, and demand

This subsection offers managerial insights by comparing the optimal results under the two subsidy policies. The following proposition summarizes the impact of government subsidy programs on price and total demand. Note that the results that we present in this paper are predicated on several threshold values that are functions of the problem parameters, primarily driven by α and *m*. These two parameters are chosen because they most directly capture the influence of consumer composition and firm's quality investment on government subsidy decisions, and therefore carry the clearest managerial implications. A dedicated summary table in Supplementary Appendix S1 that lists all these threshold expressions.

**Proposition 3**. *Comparing the prices and total demands, we have:*

*(1) when θ_1_ ≤ 1.45θ_2_, if* *m*>*m*_3_ *and α < α_1_, then* *p*^*C*^>*p*^*S*^ *and* *D*^*C*^>*D*^*S*^; *otherwise,* *p*^*C*^ ≤ *p*^*S*^ *and* *D*^*C*^ ≤ *D*^*S*^.

*(2) when 1.45θ_2_ < θ_1_ ≤ 4θ_2_, if* *m*>*m*_4_ *and α < α_1_ or *m* ≤ *m*_4_, then* *p*^*C*^>*p*^*S*^ *and *D*^*C*^>*D*^*S*^; otherwise,* *p*^*C*^ ≤ *p*^*S*^ *and* *D*^*C*^ ≤ *D*^*S*^.

*(3) when θ_1_>4θ_2_*, *p*^*C*^>*p*^*S*^ *and* *D*^*C*^>*D*^*S*^.

Proposition 3 states that under different subsidy policies, the service price of older adult care and the total demand move in the same direction, and both are influenced by the price sensitivity coefficient and market size of economy-conscious consumers, and the firm's service cost coefficient.

Specifically, when the price sensitivity coefficient of economy-conscious consumers is extremely large (θ_1_ > 4θ_2_), both the service price and total demand under the consumer subsidy policy are consistently higher than those under the service subsidy policy. This counter-intuitive finding can be explained as follows. Under the service subsidy policy, government subsidies encourage firms to improve service quality, but they also raise service prices and reduce total demand. Under the consumer subsidy policy, although firms increase service prices, which raises the expenditure of quality-conscious consumers, the government subsidy is directly provided to economy-conscious consumers, thereby lowering their effective expenditure (i.e., *p*^*C*^ − *t*^*C*^ < *p*^*S*^). Given the extremely high price sensitivity of economy-conscious consumers, even a small increase or decrease in the service price leads to a substantial drop or rise in demand. Consequently, under the consumer subsidy policy, the service price increases, yet total demand still rises.

When the price sensitivity coefficient of economy-conscious consumers is low, the service price and total demand for older adult care under different scenarios are affected by the firm's service cost coefficient and the market size of economy-conscious consumers. A high service cost implies that firms can hardly stimulate demand by improving the quality of older adult care services. In this case, if the market share of economy-conscious consumers falls below a certain threshold, firms under the consumer subsidy policy set a higher service price, which in turn generates greater total demand.

**Figure 1:**
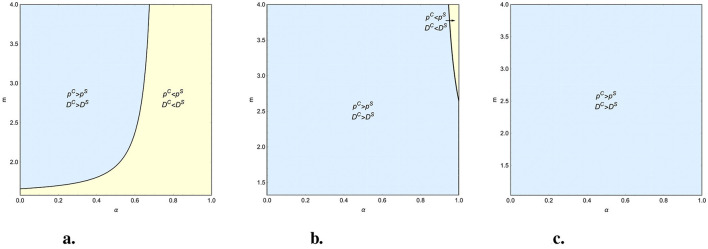
Comparison of price and total demand. **(a)** *θ*_1_ *<* 1.45*θ*_2_. **(b)** 1.45*θ*_2_ *<* *θ*_1_ *<* 4*θ*_2_. **(c)** *θ*_1_* > *4*θ*_2_.

To illustrate Proposition 3 more intuitively, we conduct numerical experiments, as shown in Figure 1. The parameter values used in the numerical experiments are illustrative and selected to satisfy the regularity conditions of the model while spanning the qualitatively distinct regimes identified in the propositions; they are not calibrated to a specific empirical dataset. Figures 1a, b indicate that when θ_1_ < 1.45θ_2_ and 1.45θ_2_ < θ_1_ < 4θ_2_, if m is large and α is small, both the service price and total demand under the consumer subsidy policy are higher. Moreover, as θ_1_ increases, the region in which the service price and total demand are both higher under the service subsidy policy gradually shrinks. In particular, when θ_1_>4θ_2_, the service price and total demand under the consumer subsidy policy are always higher, as shown in Figure 1c.

**Proposition 4**. *Comparing the service quality, we have:*

*(1) When θ_1_ ≤ 1.45θ_2_, if (i)* *m* ≤ *m*_5_, *s*^*C*^ ≤ *s*^*S*^; *(ii)* *m*>*m*_5_ *and α≥α_2_, *s*^*C*^ ≤ *s*^*S*^; otherwise,* *s*^*C*^>*s*^*S*^.

*(2) When 1.45θ_2_ < θ_1_ ≤ 1.47θ_2_, if (i)* *m* ≤ *m*_6_, *s*^*C*^≥*s*^*S*^; *(ii)* *m*_6_ ≤ *m*<*m*_7_ or *m*>*m*_8_ *and α≥α_2_, *s*^*C*^ ≤ *s*^*S*^; otherwise,* *s*^*C*^ ≤ *s*^*S*^; *(iii) if* *m*_7_ ≤ *m* ≤ *m*_8_, *s*^*C*^ ≤ *s*^*S*^.

*(3) When 1.47θ_2_ < θ_1_ ≤ 16θ_2_, if (i)* *m*<*m*_6_, *s*^*C*^>*s*^*S*^; *(ii)* *m*≥*m*_6_ *and α < α_2_*, *s*^*C*^>*s*^*S*^; otherwise, *s*^*C*^>*s*^*S*^.

*(4) When θ_1_>16θ_2_*, *s*^*C*^>*s*^*S*^.

**Figure 2:**
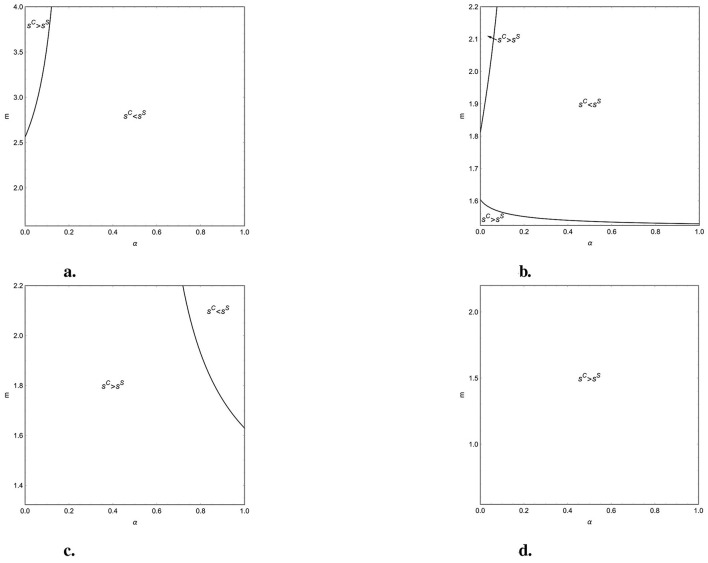
Comparison of service quality. **(a)** *θ*_1_ *<* 1.45*θ*_2_. **(b)** 1.45*θ*_2_ *< θ*_1_ *<* 1.47*θ*_2_. **(c)** 1.47*θ*_2_ *< θ*_1_ *<* 16*θ*_2_. **(d)** *θ*_1_*>* 16*θ*_2_.

Intuitively, a service subsidy policy directly incentivizes firms to improve their service quality. However, Proposition 4 reveals a non-straightforward observation: the service subsidy policy does not always lead to a higher service quality compared with the consumer subsidy policy. We use Figure 2 to visually illustrate Proposition 4.

As shown in Proposition 4(1) and Figure 2a, when the price sensitivity coefficient of economy-conscious consumers is low, and both the firm's service cost coefficient and the proportion of economy-conscious consumers in the market are low, the service quality set by the firm under the consumer-subsidy policy is actually higher than that under the service subsidy policy. A very low price sensitivity coefficient implies that the consumer subsidy policy has a minimal impact on stimulating demand. In this case, a firm with a service cost advantage will provide higher service quality under the service subsidy policy. However, when the service cost is high, the firm's incentive to improve service quality remains low under either subsidy policy. Moreover, when the proportion of economy-conscious consumers is low, the consumer subsidy policy has limited effectiveness, compelling the firm to offer higher service quality to sustain its overall profitability. As the price sensitivity coefficient increases, a firm with a cost advantage will provide higher service quality under the consumer subsidy policy, as depicted in Figures 2b, c. This is because the consumer subsidy policy begins to play a more prominent role in stimulating consumer demand.

In particular, when the price sensitivity coefficient of economy-conscious consumers is very large, the consumer subsidy policy effectively stimulates demand from this consumer segment. As stated in Proposition 3, under the consumer subsidy policy, the firm always sets a higher service price and, in turn, always provides higher service quality to meet the needs of quality-conscious consumers, as illustrated in Proposition 4(4) and Figure 2d. This implies that consumer subsidies, when well-targeted, can simultaneously advance affordability and quality, which are two pillars of the WHO Active Aging framework, without necessarily entailing a trade-off between the two.

**Figure 3:**
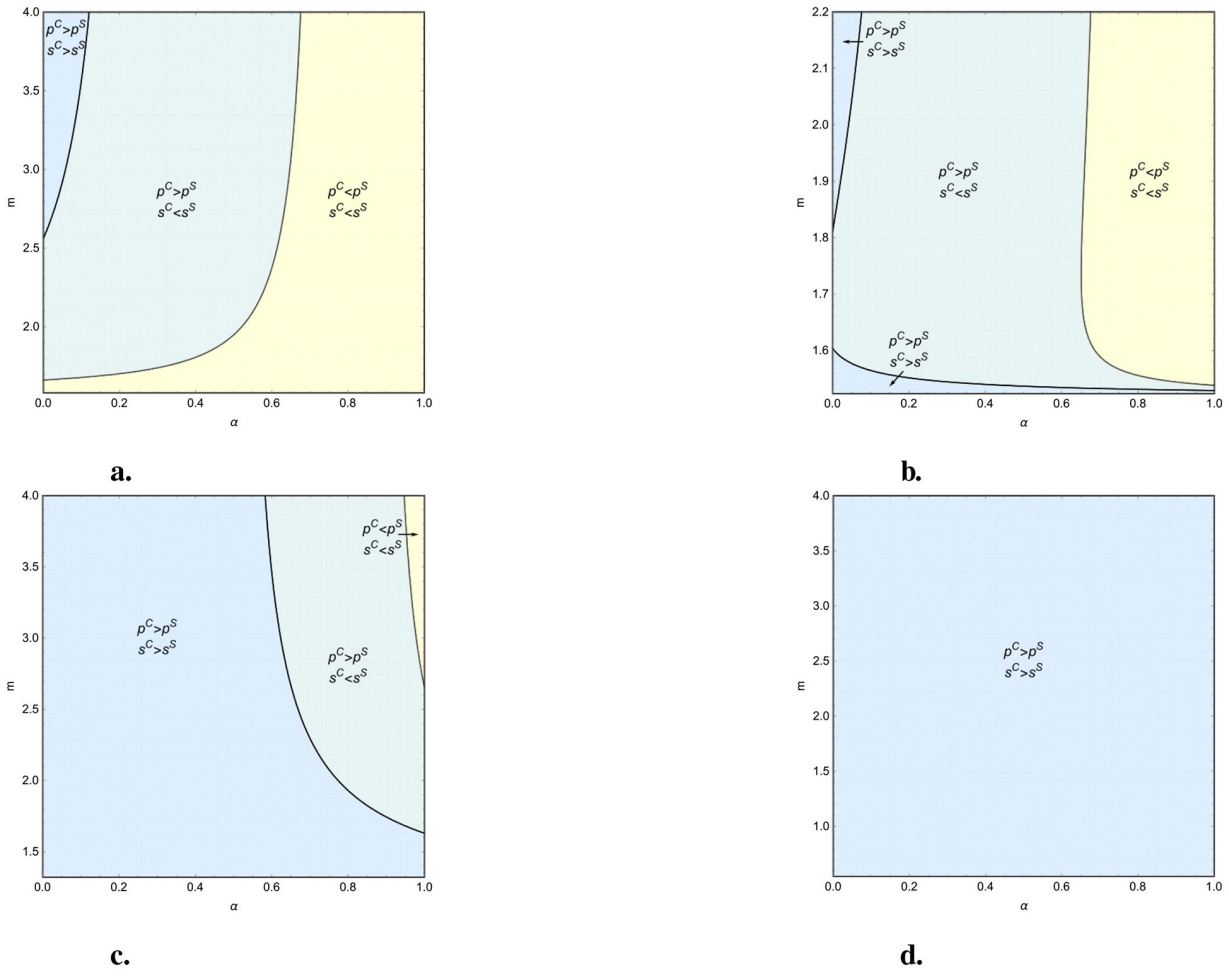
Comparison of service price and quality. **(a)** *θ*_1_ *<* 1.45*θ*_2_. **(b)** 1.45*θ*_2_ *< θ*_1_ *<* 1.47*θ*_2_. **(c)** 1.47*θ*_2_ *< θ*_1_ *<* 16*θ*_2_. **(d)** *θ*_1_ *>* 16*θ*_2_.

Next, we compare Proposition 3 and Proposition 4 through numerical analysis, with the results presented in Figure 3. The following findings can be derived from Figure 3.

First, when the price sensitivity coefficient of economy-conscious consumers is extremely low, three regions exist: (1) Blue region: the service cost is extremely high, and the market share of economy-conscious consumers is low. In this region, the consumer subsidy policy simultaneously increases both the service price and the service quality. (2) Green region: the service cost is relatively high, and the market share of economy-conscious consumers is moderate. In this region, under the consumer subsidy policy, the firm sets a higher service price but offers a lower service quality. (3) Yellow region: the market share of economy-conscious consumers is high. In this region, the consumer-subsidy policy leads the firm to reduce both the service price and the service quality. Second, as the price sensitivity coefficient of economy-conscious consumers gradually increases, the blue region expands, while both the green and yellow regions shrink. When the price sensitivity coefficient of economy-conscious consumers becomes extremely large, only the blue region remains.

### 3.2 Optimal choice of subsidy policy

We next examine the optimal subsidy policy choice from the perspectives of firm profit and social welfare, respectively, leading to the following propositions.

**Proposition 5**. *For the firm's profit:*

*(1) when θ_1_ ≤ 1.45θ_2_, if* *m*≥*m*_9_ *and α < α_3_, π^*C*^>π^*S*^; otherwise,* π^*C*^ ≤ π^*S*^.

*(2) when 1.45θ_2_ < θ_1_ ≤ 4θ_2_, if* *m*≥*m*_10_ *and α>α_3_, π^*C*^ < π^*S*^; otherwise,* π^*C*^≥π^*S*^.

*(3) when θ_1_>4θ_2_, π^*C*^>π^*S*^.*

**Figure 4:**
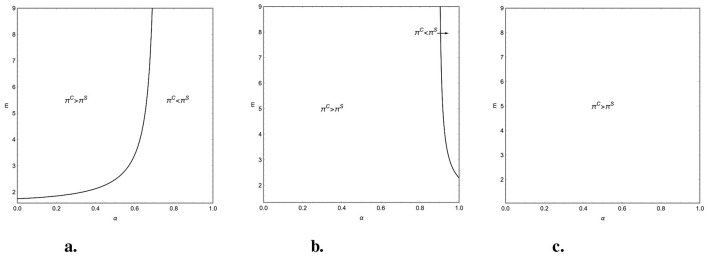
Comparison of the firm's profit. **(a)** *θ*_1_ *<* 1.45*θ*_2_. **(b)** 1.45*θ*_2_ *< θ*_1_ *<* 4*θ*_2_. **(c)** *θ*_1_ *>* 4*θ*_2_.

Proposition 5 and Figure 4 illustrate that a firm's preference for a particular subsidy policy depends on the price sensitivity coefficient of economy-conscious consumers, the service cost, and the market share of such consumers.

Specifically, when economy-conscious consumers exhibit a low price sensitivity coefficient, direct consumer subsidies have a limited effect on stimulating demand. In this case, under a low service cost, the firm is always better off under the service subsidy policy than under the consumer subsidy policy, because the former directly alleviates service cost pressure. However, when the service cost is relatively high, the firm must take into account the market share of economy-conscious consumers. As noted in Proposition 3, when this market share is low, the consumer subsidy policy enables the firm to set a higher service price and achieve higher demand, thereby generating greater profits for the firm. Conversely, when the market share is high, the service subsidy policy yields higher profits, as shown in Figure 4a.

As the price sensitivity coefficient of economy-conscious consumers gradually increases, direct consumer subsidies become increasingly effective at boosting demand. When the service cost is low, the gains in demand and profits from subsidizing consumers far outweigh the savings from reduced service costs. Consequently, the firm consistently earns higher profits under the consumer subsidy policy, as depicted in Figure 4b. Similarly, when the service cost is high and the market share of economy-conscious consumers is low, the firm is better off under the service subsidy policy.

However, when the price sensitivity coefficient of economy-conscious consumers is extremely high, the firm always prefers the consumer subsidy policy, as shown in Figure 4c. The driver of this result is that with an extremely high price sensitivity coefficient, direct consumer subsidies significantly increase consumer demand, raise both the service price and service quality, and thus generate higher revenues.

**Proposition 6**. *For the social welfare:*

*(1) when θ_1_ ≤ 1.45θ_2_, (i) if* *m* ≤ *m*_12_, *SW*^*C*^ ≤ *SW*^*S*^; *(ii) if* *m*_12_<*m* ≤ *m*_13_ *and α≥α_4_, *SW*^*C*^ ≤ *SW*^*S*^; otherwise,* *SW*^*C*^>*SW*^*S*^; *(iii) if* *m*>*m*_13_ *and α_4_ ≤ α ≤ α_5_, *SW*^*C*^ ≤ *SW*^*S*^; otherwise,* *SW*^*C*^>*SW*^*S*^.

*(2) when 1.45θ_2_ < θ_1_ ≤ 4θ_2_, (i) if* *m* ≤ *m*_14_, *SW*^*C*^≥*SW*^*S*^; *(ii) if* *m*_14_ ≤ *m* ≤ *m*_13_ *and α≥α_4_, *SW*^*C*^<*SW*^*S*^; otherwise,* *SW*^*C*^≥*SW*^*S*^; *(iii) if* *m*>*m*_13_ *and α*_4_ ≤ α ≤ α_5_, *SW*^*C*^<*SW*^*S*^; *otherwise,* *SW*^*C*^≥*SW*^*S*^.

*(3) when 4θ_2_ < θ_1_ ≤ 4.82θ_2_, (i) if* *m* ≤ *m*_14_, *SW*^*C*^≥*SW*^*S*^; *(ii) if* *m*_14_ ≤ *m* ≤ *m*_13_ *and α ≤ α_4_, *SW*^*C*^≥*SW*^*S*^; otherwise,* *SW*^*C*^<*SW*^*S*^; *(iii) if* *m*>*m*_13_, *SW*^*C*^>*SW*^*S*^.

*(4) when θ_1_>4.82θ_2_, *SW*^*C*^>*SW*^*S*^*.

**Figure 5:**
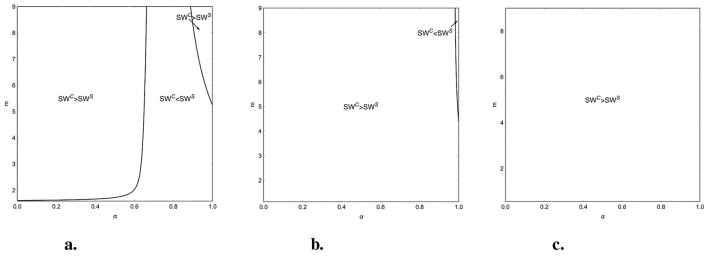
Comparison of the social welfare. **(a)** *θ*_1_ *<* 4*θ*_2_. **(b)** 4*θ*_2_ *< θ*_1_ *<* 4.82*θ*_2_. **(c)** *θ*_1_ *>* 4.82*θ*_2_.

Both consumer subsidy and service subsidy policies are widely adopted by governments to stimulate sales and improve social welfare. Nevertheless, which subsidy scheme is more beneficial to social welfare remains unclear. Proposition 6 and Figure 5 show that the government's optimal subsidy policy similarly depends on the price sensitivity coefficient of economy conscious consumers, the service cost, and the market share of such consumers.

Figure 5a indicates that in markets where economy conscious consumers have a relatively low price sensitivity coefficient, the government prefers the consumer subsidy policy when the service cost is high and the market share of economy conscious consumers is either low or high; otherwise, the firm subsidy policy is superior. Figure 5b shows that in markets with a moderate price sensitivity coefficient, the government's optimal choice is the service subsidy policy when both the service cost and the market share of economy conscious consumers are large. Figure 5c reveals that in markets with an extremely high price sensitivity coefficient of economy conscious consumers, it is optimal for the government to subsidize economy conscious consumers, as this consistently yields higher social welfare.

Thus, it is evident that the price sensitivity coefficient of economy conscious consumers plays a critical role in the government's choice of subsidy policy. As this coefficient increases, the region in which the consumer subsidy policy generates greater social welfare expands. In other words, the government should lean toward the consumer subsidy policy.

This finding has important equity implications. Given the high price sensitivity of low-income older adult individuals, consumer subsidies that directly reduce out-of-pocket costs are more effective than service subsidies in expanding their participation in the care market. Because service subsidies are only partially passed through to consumers and may be offset by providers' price increases, governments seeking to reduce income-based disparities in long-term care access should prioritize consumer subsidies, particularly when low-income older adult individuals constitute a large share of the target population.

**Figure 6:**
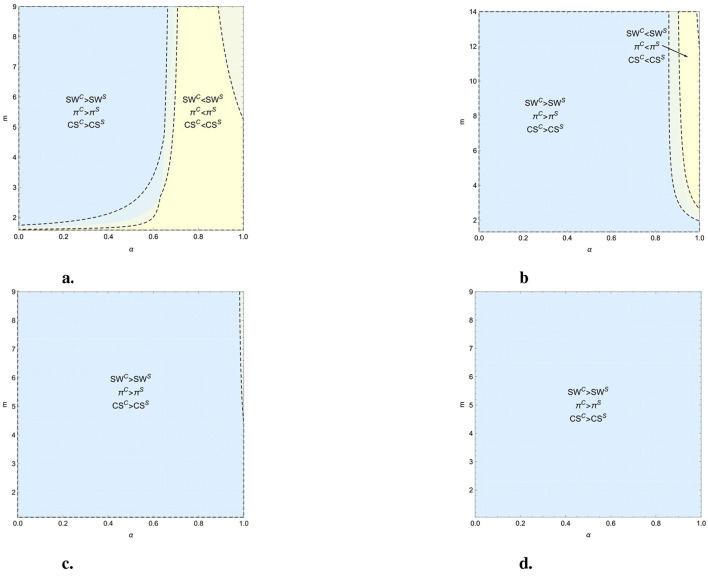
Comparison of the consumer surplus, firm's profit and social welfare. **(a)** *θ*_1_ *<* 1.45*θ*_2_. **(b)** 1.45*θ*_2_ *<* *θ*_1_ *<* 4*θ*_2_. **(c)** 4*θ*_2_ *< θ*_1_ *<* 4.82*θ*_2_. **(d)** *θ*_1_ *>* 4.82*θ*_2_.

Thus far, we have examined which subsidy policy is more favorable from the perspectives of consumers, firm, and the government individually. To further investigate which policy can achieve a triple-win outcome for consumers, firm, and the government simultaneously, we conduct numerical experiments, with results shown in Figure 6.

The following findings emerge from Figure 6. First, when the price sensitivity coefficient of economy conscious consumers is relatively low, both consumer subsidy and service subsidy can realize a triple-win outcome, as illustrated in Figures 6a, b. In the blue (yellow) region, consumer subsidy (service subsidy) yields higher consumer surplus, firm profit, and social welfare. Second, when the price sensitivity coefficient of economy conscious consumers is relatively high, only consumer subsidy achieves a triple-win outcome across most of the parameter space, as shown in Figures 6c, d.

## 4 Conclusion

In this study, we establish an older adult care service supply chain model incorporating subsidy policies to address the decision-making problems of the firm and the government. The government faces a binary choice regarding the subsidy policy: a consumer subsidy policy or a service subsidy policy. Under a given subsidy policy, the firm sells older adult care services to two types of consumers: economy conscious consumers and quality conscious consumers. We develop two models to analyze the different subsidy policies from the perspectives of consumers, the firm, and the government. Our study yields the following main conclusions.

First, from the perspective of social welfare, the government's optimal choice of subsidy policy is primarily influenced by the price sensitivity coefficient of economy-conscious consumers, the service cost, and the market share of such consumers. Specifically, when the price sensitivity coefficient of economy-conscious consumers is low, the consumer subsidy policy generates higher social welfare if the service cost is high and the market share of economy conscious consumers is either sufficiently low or sufficiently high; otherwise, the service subsidy policy should be adopted. As the price sensitivity coefficient increases, the consumer subsidy policy exhibits an increasingly pronounced advantage in enhancing social welfare.

Second, from the perspective of firm profit, which subsidy policy yields higher profits also depends on the price sensitivity coefficient of economy conscious consumers, the service cost, and their market share. Specifically, when the price sensitivity coefficient is low, the firm prefers the consumer subsidy policy if the service cost is high and the market share of economy conscious consumers is low; otherwise, the service subsidy policy is superior. When the price sensitivity coefficient is moderate, the firm prefers the service subsidy policy if both the service cost and the market share of economy conscious consumers are high; otherwise, the consumer subsidy policy is better. However, when the price sensitivity coefficient is high, the consumer subsidy policy always dominates the service subsidy policy.

Third, from the perspective of consumers, the consumer subsidy policy may in-deed induce the firm to raise prices, especially when the price sensitivity coefficient of economy conscious consumers is high. Although the service price increases to some extent under the consumer subsidy policy, the firm also improves service quality, leading to higher consumer demand and consumer surplus. Moreover, the service subsidy policy does not necessarily guarantee higher service quality than the consumer subsidy policy.

Fourth, when the price sensitivity coefficient of economy conscious consumers is low, both the consumer subsidy policy and the service subsidy policy can achieve a tripartite win-win situation for consumers, the firm, and the government. However, when the price sensitivity coefficient is high, only the consumer subsidy policy can realize such a tripartite outcome.

Beyond economic efficiency, our findings carry direct public health implications. The consumer subsidy policy, when economy-conscious consumers exhibit high price sensitivity, simultaneously achieves superior consumer surplus, firm profit, and social welfare. By directly reducing out-of-pocket costs for low-income older adult individuals, it lowers financial barriers to care access and promotes health equity. Importantly, this improvement in affordability does not necessarily come at the expense of service quality, because high price sensitivity encourages firms to enhance quality in response to increased demand, while sustained profitability helps maintain the long-term viability of the care supply chain. Governments should therefore treat the price sensitivity profile of the older adult population as a central input in subsidy design, as it simultaneously determines the economically optimal policy and the instrument best aligned with public health objectives.

This research could be extended in several directions. First, this paper only considers a market with a single service firm. It would be interesting to extend our models to account for competition among multiple service firms and to analyze how competition in service price and quality affects the government's choice of subsidy policy. Second, we do not consider factors such as government budget constraints or asymmetric demand information. These factors merit further investigation in future research. Third, we characterize older adult consumers using two representative preference types. Future research could incorporate richer consumer heterogeneity, including differences in income, age, health status, dependency level, residence, and family support, to investigate more targeted and equitable subsidy policies. In addition, future studies could integrate explicit health outcome measures, such as hospitalization rates or quality-adjusted life years, into the social welfare framework, thereby providing a more comprehensive assessment of the long-term effectiveness of alternative subsidy schemes.

## Data Availability

The original contributions presented in the study are included in the article/Supplementary material, further inquiries can be directed to the corresponding author.

## Footnotes

1
https://english.news.cn/20260324/4c3bb198970b4129854f7962f1920db8/c.html

2
https://english.www.gov.cn/policies/latestreleases/202401/16/content_WS65a5b709c6d0868f4e8e31cc.html
