# Supplementary material for: Subsidize consumers or service firm? The design of a government subsidy policy for an older adult care service supply chain
Source: Front Public Health. 2026 Jul 22;14:1890740. doi: 10.3389/fpubh.2026.1890740 (PMC13437359; doi:10.3389/fpubh.2026.1890740)
Supplement: Supplementary file 1 [file Data_Sheet_1.docx]

**Subsidize consumers or service firm? The design of a government subsidy policy for an** **elderly care service supply chain**

# Appendix

**Proof of Lemma 1 and Proposition 1.** Since $\frac{\partial^{2}\pi^{C}}{\partial p^{2}}=-2(\theta_{1}+\theta_{2})<0$, we can get the best service price response function as $p=\frac{M+2s+t\theta_{1}}{2\left( \theta_{1}+\theta_{2} \right)}$. We plug this function into $\pi^{C}$ and further take the first-order derivative of $\pi^{C}$ with respect to $s$ to obtain the best service quality response function as $s=\frac{M+t\theta_{1}}{m\left( \theta_{1}+\theta_{2} \right)-2}$ because of $\frac{\partial^{2}\pi^{C}}{\partial s^{2}}=\frac{3}{\theta_{1}+\theta_{2}}-m<0$, where $m>\{\frac{8}{3\theta_{1}+6\theta_{2}-\sqrt{5\theta_{1}^{2}+4\theta_{2}^{2}}},\frac{3}{\theta_{1}+\theta_{2}}\}$. Substituting $s=\frac{M+t\theta_{1}}{m\left( \theta_{1}+\theta_{2} \right)-2}$ into $p=\frac{M+2s+t\theta_{1}}{2\left( \theta_{1}+\theta_{2} \right)}$ can obtain $p=\frac{m\left( M+t\theta_{1} \right)}{2m\left( \theta_{1}+\theta_{2} \right)-4}$.

Then, we take the best response prices $p$ and $s$ into $SW^{C}$ and obtain the second-order derivative of $SW^{C}$ with respect to $t$ as $\frac{\partial^{2}SW^{C}}{\partial t^{2}}=-\frac{\theta1\left( 16-12m\left( \theta_{1}+2\theta_{2} \right)+m^{2}\left( \theta_{1}+\theta_{2} \right)\left( \theta_{1}+8\theta_{2} \right) \right)}{4\left( m\left( \theta_{1}+\theta_{2} \right)-2 \right)^{2}}<0$. Then, the optimal per-unit consumer subsidy of government is $t^{C}=\frac{M(8+5m^{2}\theta_{1}(\theta_{1}+\theta_{2})-4m(3\theta_{1}+\theta_{2})-4\alpha{(2-m(\theta_{1}+\theta_{2}))}^{2})}{\theta_{1}(16-12m\left( \theta_{1}+2\theta_{2} \right)+m^{2}\left( \theta_{1}+\theta_{2} \right)\left( \theta_{1}+8\theta_{2} \right))}$. As a result, taking $t^{C}$ into the firm’s best price and quality response functions can get $p^{C}$ and $s^{C}$. Lastly, taking these optimal functions into demand, profit and social welfare functions yields the optimal outcomes.

**Proof of Lemma 2 and Proposition 2.** The proof is similar to that of Lemma 1 and Proposition 1.

**Proof of Proposition 3.** If $\theta_{1}>1.45\theta_{2}$, $\frac{8}{3\theta_{1}+6\theta_{2}-\sqrt{5\theta_{1}^{2}+4\theta_{2}^{2}}}>\frac{3}{\theta_{1}+\theta_{2}}$; else if $\theta_{1}\leq1.45\theta_{2}$, $\frac{8}{3\theta_{1}+6\theta_{2}-\sqrt{5\theta_{1}^{2}+4\theta_{2}^{2}}}\leq\frac{3}{\theta_{1}+\theta_{2}}$. Then, when $\theta_{1}>1.45\theta_{2}$, $m>m_{1}$, otherwise, $m>m_{2}$.

Comparing $p^{C}$ with $p^{S}$. As $\frac{\partial\left( p^{C}-p^{S} \right)}{\partial\alpha}=\frac{mM\left( 4-2m\left( \theta1+\theta2 \right) \right)}{16-12m\left( \theta1+2\theta2 \right)+m^{2}\left( \theta1+\theta2 \right)\left( \theta1+8\theta2 \right)}<0$ when $m>max\{m_{1},m_{2}\}$; and (1) when $\theta_{1}\leq1.45\theta_{2}$, $p^{C}-p^{S}|_{\alpha=0}\leq0$ if $m_{2}<m\leq m_{3}$, $p^{C}-p^{S}|_{\alpha=0}>0$ if $m>m_{3}$, and $p^{C}-p^{S}|_{\alpha=1}<0$; (2) $1.45\theta_{2}<\theta_{1}\leq4\theta_{2}$, $p^{C}-p^{S}|_{\alpha=0}>0$, and $p^{C}-p^{S}|_{\alpha=1}>0$ if $m_{1}<m<m_{4}$, $p^{C}-p^{S}|_{\alpha=1}\leq0$ if $m>m_{4}$; (3) $\theta_{1}>4\theta_{2}$, $p^{C}-p^{S}|_{\alpha=0}>0$ and $p^{C}-p^{S}|_{\alpha=1}>0$.

To summarize, (1) when $\theta_{1}\leq1.45\theta_{2}$, if $m\leq m_{3}$, $p^{C}\leq p^{S}$; else if $m>m_{3}$ and $\alpha<\alpha_{1}$, $p^{C}>p^{S}$; else if $m>m_{3}$ and $\alpha\geq\alpha_{1}$, $p^{C}\leq p^{S}$. (2) When $1.45\theta_{2}<\theta_{1}\leq4\theta_{2}$, if $m<m_{4}$, $p^{C}>p^{S}$; else if $m\geq m_{4}$ and $\alpha<\alpha_{1}$, $p^{C}>p^{S}$; else if $m\geq m_{4}$ and $\alpha\geq\alpha_{1}$, $p^{C}\leq p^{S}$. (3) When $\theta_{1}>4\theta_{2}$, $p^{C}>p^{S}$.

Comparing $D^{C}$ with $D^{S}$ the proof is similar to that of $p^{C}$ and $p^{S}$.

**Proof of Proposition 4.** Comparing $s^{C}$ and $s^{S}$, the proof is similar to that of Proposition 3.

**Proof of Proposition 5.** Comparing $\pi^{C}$ with $\pi^{S}$, the proof is similar to that of Proposition 3.

**Proof of Proposition 6.** Comparing ${SW}^{C}$ with ${SW}^{S}$. As $\frac{\partial^{2}({SW}^{C}-{SW}^{S})}{\partial\alpha^{2}}=\frac{4M^{2}\left( -2+m\left( \theta_{1}+\theta_{2} \right) \right)^{2}}{\theta_{1}\left( 16-12m\left( \theta_{1}+2\theta_{2} \right)+m^{2}\left( \theta_{1}+\theta_{2} \right)\left( \theta_{1}+8\theta_{2} \right) \right)}>0$; The axis of symmetry of ${SW}^{C}-{SW}^{S}$ is

$\frac{8+5m^{2}\theta_{1}\left( \theta_{1}+\theta_{2} \right)-4m\left( 3\theta_{1}+\theta_{2} \right)}{4\left( -2+m\left( \theta_{1}+\theta_{2} \right) \right)^{2}}>0$, ${SW}^{C}-{SW}^{S}|_{\alpha=\frac{8+5m^{2}\theta_{1}\left( \theta_{1}+\theta_{2} \right)-4m\left( 3\theta_{1}+\theta_{2} \right)}{4\left( -2+m\left( \theta_{1}+\theta_{2} \right) \right)^{2}}}=\frac{-m^{2}M^{2}\left( \theta1+\theta2 \right)}{8\left( m\left( \theta1+\theta2 \right)-3 \right)\left( m\left( \theta1+\theta2 \right)-2 \right)^{2}}<0$, and $\frac{8+5m^{2}\theta_{1}\left( \theta_{1}+\theta_{2} \right)-4m\left( 3\theta_{1}+\theta_{2} \right)}{4\left( -2+m\left( \theta_{1}+\theta_{2} \right) \right)^{2}}\geq1$ if $\theta_{1}\geq4\theta_{2}$, or $\theta_{1}<4\theta_{2}$ and $m\leq m_{11}$; otherwise, $\frac{8+5m^{2}\theta_{1}\left( \theta_{1}+\theta_{2} \right)-4m\left( 3\theta_{1}+\theta_{2} \right)}{4\left( -2+m\left( \theta_{1}+\theta_{2} \right) \right)^{2}}<1$ if $\theta_{1}<4\theta_{2}$ and $m>m_{11}$.

Then, identify the boundary values of ${SW}^{C}-{SW}^{S}$. (1) $\theta_{1}\leq1.45\theta_{2}$, ${SW}^{C}-{SW}^{S}|_{\alpha=0}>0$ if $m>m_{12}$, ${SW}^{C}-{SW}^{S}|_{\alpha=0}\leq0$ if $m\leq m_{12}$, and ${SW}^{C}-{SW}^{S}|_{\alpha=1}>1$ if $m>m_{13}$, ${SW}^{C}-{SW}^{S}|_{\alpha=0}\leq1$ if $m\leq m_{13}$; (2) $1.45\theta_{2}<\theta_{1}\leq4\theta_{2}$, ${SW}^{C}-{SW}^{S}|_{\alpha=0}>0$, and ${SW}^{C}-{SW}^{S}|_{\alpha=1}>1$ if $m>m_{13}$ or $m<m_{14}$, ${SW}^{C}-{SW}^{S}|_{\alpha=0}\leq1$ if $m_{14}<m\leq m_{13}$; (3) $4\theta_{2}\leq\theta_{1}<4.82\theta_{2}$, ${SW}^{C}-{SW}^{S}|_{\alpha=0}>0$, and ${SW}^{C}-{SW}^{S}|_{\alpha=1}>1$ if $m>m_{13}$ or $m<m_{14}$, ${SW}^{C}-{SW}^{S}|_{\alpha=0}\leq1$ if $m_{14}<m\leq m_{13}$; (4) $\theta_{1}\geq4.82\theta_{2}$, ${SW}^{C}-{SW}^{S}|_{\alpha=0}>0$, and ${SW}^{C}-{SW}^{S}|_{\alpha=1}>0$.

To summarize, (1) when $\theta_{1}\leq1.45\theta_{2}$, if $m\leq m_{12}$, ${SW}^{C}\leq{SW}^{S}$; else if $m_{12}<m\leq m_{13}$ and $\alpha<\alpha_{4}$, ${SW}^{C}>{SW}^{S}$; else if $m_{12}<m\leq m_{13}$ and $\alpha\geq\alpha_{4}$, ${SW}^{C}\leq{SW}^{S}$; else if $m>m_{13}$ and $\alpha<\alpha_{4}$ or $m>m_{13}$ and $\alpha>\alpha_{5}$, ${SW}^{C}>{SW}^{S}$; else if $m>m_{13}$ and $\alpha_{4}\leq\alpha\leq\alpha_{5}$, ${SW}^{C}\leq{SW}^{S}$. (2) When $1.45\theta_{2}<\theta_{1}\leq4\theta_{2}$, if $m<m_{14}$, ${SW}^{C}>{SW}^{S}$; else if $m_{14}\leq m\leq m_{13}$ and $\alpha<\alpha_{4}$, ${SW}^{C}>{SW}^{S}$; else if $m_{14}\leq m\leq m_{13}$ and $\alpha\geq\alpha_{4}$, ${SW}^{C}\leq{SW}^{S}$; else if $m>m_{13}$ and $\alpha<\alpha_{4}$ or $m>m_{13}$ and $\alpha>\alpha_{5}$, ${SW}^{C}>{SW}^{S}$; else if $m>m_{13}$ and $\alpha_{4}\leq\alpha\leq\alpha_{5}$, ${SW}^{C}\leq{SW}^{S}$. (3) When $4\theta_{2}<\theta_{1}\leq4.82\theta_{2}$, if $m<m_{14}$, ${SW}^{C}>{SW}^{S}$; else if $m_{14}\leq m\leq m_{13}$ and $\alpha<\alpha_{4}$, ${SW}^{C}>{SW}^{S}$; else if $m_{14}\leq m\leq m_{13}$ and $\alpha\geq\alpha_{4}$, ${SW}^{C}\leq{SW}^{S}$; else if $m>m_{13}$, $\theta_{1}\leq4.82\theta_{2}$. (4) When $\theta_{1}>4.82\theta_{2}$, ${SW}^{C}>{SW}^{S}$.

**Table of parameter thresholds.**

| $m_{1}$ | $8/(3\theta_{1}+6\theta_{2}-\sqrt{5\theta_{1}^{2}+4\theta_{2}^{2}})$. |
| --- | --- |
| $m_{2}$ | $\frac{3}{\theta_{1}+\theta_{2}}$. |
| $m_{3}$ | $\frac{9\theta_{1}+6\theta_{2}+\sqrt{36\theta_{2}^{2}+8\theta_{1}\theta_{2}-19\theta_{1}^{2}}}{5\theta_{1}^{2}+5\theta_{1}\theta_{2}}$. |
| $m_{4}$ | $\frac{4}{\theta_{1}-\sqrt{\theta_{1}\left( 5\theta_{1}-4\theta_{2} \right)}+4\theta_{2}}$. |
| $m_{5}$ | $\frac{12}{6\theta_{1}-\sqrt{6}\sqrt{8\theta_{2}^{2}-3\theta_{1}^{2}-\theta_{1}\theta_{2}}}$. |
| $m_{6}$ | $\frac{12}{4\theta_{1}+10\theta_{2}-\sqrt{22\theta_{1}^{2}-10\theta_{1}\theta_{2}+4\theta_{2}^{2}}}$. |
| $m_{7}$ | $\frac{2\left( 6\theta_{1}-\sqrt{6}\sqrt{8\theta_{2}^{2}-3\theta_{1}^{2}-\theta_{1}\theta_{2}} \right)}{9\theta_{1}^{2}+\theta_{1}\theta_{2}-8\theta_{2}^{2}}$. |
| $m_{8}$ | $\frac{2\left( 6\theta_{1}+\sqrt{6}\sqrt{8\theta_{2}^{2}-3\theta_{1}^{2}-\theta_{1}\theta_{2}} \right)}{9\theta_{1}^{2}+\theta_{1}\theta_{2}-8\theta_{2}^{2}}$. |
| $m_{9}$ | $Root[-4416+\left( 9568\theta_{1}+10144\theta_{2} \right)\#1+\left( -8352\theta_{1}^{2}-17280\theta_{1}\theta_{2}-8640\theta_{2}^{2} \right){\#1}^{2}+\left( 3680\theta_{1}^{3}+11216\theta_{1}^{2}\theta_{2}+10768\theta_{1}\theta_{2}^{2}+3232\theta_{2}^{3} \right){\#1}^{3}+\left( -813\theta_{1}^{4}-3306\theta_{1}^{3}\theta_{2}-4621\theta_{1}^{2}\theta_{2}^{2}-2576\theta_{1}\theta_{2}^{3}-448\theta_{2}^{4} \right){\#1}^{4}+\left( 70\theta_{1}^{5}+370\theta_{1}^{4}\theta_{2}+690\theta_{1}^{3}\theta_{2}^{2}+550\theta_{1}^{2}\theta_{2}^{3}+160\theta_{1}\theta_{2}^{4} \right){\#1}^{5}\&,3]$. |
| $m_{10}$ | $Root[192+\left( -416\theta_{1}-992\theta_{2} \right)\#1+\left( 224\theta_{1}^{2}+1792\theta_{1}\theta_{2}+1856\theta_{2}^{2} \right){\#1}^{2}+\left( 32\theta_{1}^{3}-912\theta_{1}^{2}\theta_{2}-2544\theta_{1}\theta_{2}^{2}-1600\theta_{2}^{3} \right){\#1}^{3}+\left( -45\theta_{1}^{4}+86\theta_{1}^{3}\theta_{2}+947\theta_{1}^{2}\theta_{2}^{2}+1456\theta_{1}\theta_{2}^{3}+640\theta_{2}^{4} \right){\#1}^{4}+\left( 6\theta_{1}^{5}+18\theta_{1}^{4}\theta_{2}-78\theta_{1}^{3}\theta_{2}^{2}-282\theta_{1}^{2}\theta_{2}^{3}-288\theta_{1}\theta_{2}^{4}-96\theta_{2}^{5} \right){\#1}^{5}\&,3]$. |
| $m_{11}$ | $\frac{4}{\theta_{1}+3\theta_{2}-\sqrt{3\theta_{1}^{2}+\theta_{2}^{2}}}$. |
| $m_{12}$ | $\frac{B_{1}}{3A_{1}}-\frac{\left( 1+ⅈ\sqrt{3} \right)\left( -B_{1}^{2}+3A_{1}C_{1} \right)}{3\times2^{2/3}A_{1}\left( -1296A_{1}^{2}-2B_{1}^{3}+9A_{1}B_{1}C_{1}+\sqrt{-4\left( B_{1}^{2}-3A_{1}C_{1} \right)^{3}+\left( 1296A_{1}^{2}+2B_{1}^{3}-9A_{1}B_{1}C_{1} \right)^{2}} \right)^{1/3}}+\frac{\left( 1-ⅈ\sqrt{3} \right)\left( -1296A_{1}^{2}-2B_{1}^{3}+9A_{1}B_{1}C_{1}+\sqrt{-4\left( B_{1}^{2}-3A_{1}C_{1} \right)^{3}+\left( 1296A_{1}^{2}+2B_{1}^{3}-9A_{1}B_{1}C_{1} \right)^{2}} \right)^{1/3}}{6\times2^{1/3}A_{1}}$. |
| $m_{13}$ | $-\frac{B_{2}}{3A_{2}}-\frac{2^{1/3}\left( -B_{2}^{2}+3A_{2}C_{2} \right)}{3A_{2}\left( 1296A_{2}^{2}-2B_{2}^{3}+9A_{2}B_{2}C_{2}+\sqrt{4\left( -B_{2}^{2}+3A_{2}C_{2} \right)^{3}+\left( 1296A_{2}^{2}-2B_{2}^{3}+9A_{2}B_{2}C_{2} \right)^{2}} \right)^{1/3}}+\frac{\left( 1296A_{2}^{2}-2B_{2}^{3}+9A_{2}B_{2}C_{2}+\sqrt{4\left( -B_{2}^{2}+3A_{2}C_{2} \right)^{3}+\left( 1296A_{2}^{2}-2B_{2}^{3}+9A_{2}B_{2}C_{2} \right)^{2}} \right)^{1/3}}{3{\times2}^{1/3}A_{2}}$. |
| $m_{14}$ | $-\frac{B_{2}}{3A_{2}}+\frac{\left( 1-ⅈ\sqrt{3} \right)\left( -B_{2}^{2}+3A_{2}C_{2} \right)}{3\times2^{2/3}A_{2}\left( 1296A_{2}^{2}-2B_{2}^{3}+9A_{2}B_{2}C_{2}+\sqrt{4\left( -B_{2}^{2}+3A_{2}C_{2} \right)^{3}+\left( 1296A_{2}^{2}-2B_{2}^{3}+9A_{2}B_{2}C_{2} \right)^{2}} \right)^{1/3}}-\frac{\left( 1+ⅈ\sqrt{3} \right)\left( 1296A_{2}^{2}-2B_{2}^{3}+9A_{2}B_{2}C_{2}+\sqrt{4\left( -B_{2}^{2}+3A_{2}C_{2} \right)^{3}+\left( 1296A_{2}^{2}-2B_{2}^{3}+9A_{2}B_{2}C_{2} \right)^{2}} \right)^{1/3}}{6\times2^{1/3}A_{2}}$. |
| $\alpha_{1}$ | $\frac{16-9m\theta_{1}}{4\left( 3-m\left( \theta_{1}+\theta_{2} \right) \right)}+\frac{m\theta_{1}-1}{2-m\left( \theta_{1}+\theta_{2} \right)}$. |
| $\alpha_{2}$ | $\frac{24-24m\theta_{1}+m^{2}\left( 9\theta_{1}-8\theta_{2} \right)\left( \theta_{1}+\theta_{2} \right)}{8\left( 3-m\left( \theta_{1}+\theta_{2} \right) \right)\left( 2-m\left( \theta_{1}+\theta_{2} \right) \right)}$. |
| $\alpha_{3}$ | $\frac{B_{3}-\sqrt{B_{3}^{2}-4A_{3}C_{3}}}{2A_{3}}$. |
| $\alpha_{4}$ | $\frac{B_{4}-\sqrt{B_{4}^{2}-4A_{4}C_{4}}}{2A_{4}}$. |
| $\alpha_{5}$ | $\frac{B_{4}+\sqrt{B_{4}^{2}-4A_{4}C_{4}}}{2A_{4}}$. |
| $A_{1}$ | $25\theta_{1}^{2}\left( \theta_{1}+\theta_{2} \right)$. |
| $B_{1}$ | $48\theta_{1}\left( 2\theta_{1}+\theta_{2} \right)$. |
| $C_{1}$ | $16\left( 7\theta_{1}+\theta_{2} \right)$. |
| $A_{2}$ | $\left( \theta_{1}-4\theta_{2} \right)^{2}\left( \theta_{1}+\theta_{2} \right)$. |
| $B_{2}$ | $8\left( \theta_{1}-5\theta_{2} \right)\left( \theta_{1}+2\theta_{2} \right)$. |
| $C_{2}$ | $16\left( \theta_{1}+7\theta_{2} \right)$. |
| $A_{3}$ | $\frac{m\left( -2+m\left( \theta_{1}+\theta_{2} \right) \right)\left( -4+2m\left( \theta_{1}+\theta_{2} \right) \right)^{2}}{\left( 16-12m\left( \theta_{1}+2\theta_{2} \right)+m^{2}\left( \theta_{1}+\theta_{2} \right)\left( \theta_{1}+8\theta_{2} \right) \right)^{2}}$. |
| $B_{3}$ | $\frac{2m\left( -2+m\left( \theta_{1}+\theta_{2} \right) \right)\left( -4+2m\left( \theta_{1}+\theta_{2} \right) \right)\left( -6+3m\theta_{1}+4m\theta_{2} \right)}{\left( 16-12m\left( \theta_{1}+2\theta_{2} \right)+m^{2}\left( \theta_{1}+\theta_{2} \right)\left( \theta_{1}+8\theta_{2} \right) \right)^{2}}$. |
| $C_{3}$ | $\frac{m\left( 3-2m\left( \theta_{1}+\theta_{2} \right) \right)}{8\left( -3+m\left( \theta_{1}+\theta_{2} \right) \right)^{2}}+\frac{m\left( -2+m\left( \theta_{1}+\theta_{2} \right) \right)\left( -6+3m\theta_{1}+4m\theta_{2} \right)^{2}}{\left( 16-12m\left( \theta_{1}+2\theta_{2} \right)+m^{2}\left( \theta_{1}+\theta_{2} \right)\left( \theta_{1}+8\theta_{2} \right) \right)^{2}}$. |
| $A_{4}$ | $\frac{16\left( -2+m\left( \theta_{1}+\theta_{2} \right) \right)^{2}}{\theta_{1}\left( 16-12m\left( \theta_{1}+2\theta_{2} \right)+m^{2}\left( \theta_{1}+\theta_{2} \right)\left( \theta_{1}+8\theta_{2} \right) \right)}$. |
| $B_{4}$ | $\frac{8\left( 8+5m^{2}\theta_{1}\left( \theta_{1}+\theta_{2} \right)-4m\left( 3\theta_{1}+\theta_{2} \right) \right)}{\theta_{1}\left( 16-12m\left( \theta_{1}+2\theta_{2} \right)+m^{2}\left( \theta_{1}+\theta_{2} \right)\left( \theta_{1}+8\theta_{2} \right) \right)}$. |
| $C_{4}$ | $\frac{4\left( 4+m\theta_{1}\left( -12+7m\theta_{1}+6m\theta_{2} \right) \right)}{\theta_{1}\left( 16-12m\left( \theta_{1}+2\theta_{2} \right)+m^{2}\left( \theta_{1}+\theta_{2} \right)\left( \theta_{1}+8\theta_{2} \right) \right)}-\frac{3m}{-3+m\left( \theta_{1}+\theta_{2} \right)}$. |
